# The role of phase I, phase II, and DNA-repair gene polymorphisms in the damage induced by formaldehyde in pathologists

**DOI:** 10.1038/s41598-021-89833-w

**Published:** 2021-05-18

**Authors:** Federica Ghelli, Enrico Cocchi, Martina Buglisi, Giulia Squillacioti, Valeria Bellisario, Roberto Bono, Alfredo Santovito

**Affiliations:** 1 Department of Public Health and Pediatrics, University of Turin, Via Santena 5 bis, 10126 Turin, Italy; 2 Department of Life Sciences and Systems Biology, University of Turin, Via Accademia Albertina 13, 10123 Turin, Italy

**Keywords:** Genetics, Biomarkers, Health occupations, Risk factors

## Abstract

Formaldehyde (FA) is a human carcinogen used as formalin in hospital laboratories. We evaluated its association with human chromosomal aberrations (CAs) and the risk/protective role played by several genetic polymorphisms in this relationship, on a cohort of 57 exposed pathologists *vs* 48 controls. All subjects were assessed for CAs on peripheral blood lymphocytes and genotyped for the most common cancer-associated gene polymorphisms which could be related with the genotoxic outcome: *CYP1A1 exon 7 (A*>*G)*, CYP1A1*2A (T>*C)*, CYP2C19*2 (G>*A)*, *GSTT1* (Positive/Null), *GSTM1* (Positive/null), *GSTP1 (A*>*G)*, *XRCC1 (G399A)*, *XRCC1 (C194T)*, *XRCC1 (A280G)*, *XPD (A751C)*, *XPC exon 15 (A939C)*, *XPC exon 9 (C499T)*, *TNFα − 308 (G*>*A)*, *IL10 − 1082 (G*>*A)*, *IL10 − 819 (C*>*T)* and *IL6 − 174 (G*>*C)*. Air-FA concentration was assessed through personal samplers. The comparison between pathologists and controls showed a significantly higher CAs frequency in pathologists. Significant positive correlations were found between CAs frequency and air-FA concentration while significant associations were found between variation in CAs frequency and the mutated allele for *CYP1A1 exon 7 (A*>*G)*, CYP2C19*2 (G>*A)*, *GSTT1*-positive, *GSTM1*-positive and *XRCC1 (G399A)*. Our study confirms the role of FA as genotoxicity inductor, even in workers chronically exposed to low air-FA levels and reveals the role played by some genetic polymorphisms in this association, highlighting the importance of individual susceptibility biomarkers assessment in occupational health studies.

## Introduction

Formaldehyde (FA) is a compound produced worldwide and employed in an extremely wide variety of industrial and medical processes^1^, resulting in a widespread exposure in both environmental and occupational contexts^2^. As it is well-known, FA is responsible of several biological effects, even at lower concentrations than those recommended by the American Conference of Governmental Industrial Hygienists (ACGIH)^3,4^. Workers exposed to FA are at increased risk of cancer, especially nasopharyngeal cancer and myeloid leukaemia^5^. Due to these effects, FA is classified as a group I human carcinogen by the International Agency for Research on Cancer (IARC) since 2006^5–7^; nevertheless, considerable discrepancies remain among guidelines suggested for occupational exposure to FA. The ACGIH recommended a Threshold Limit Value-Ceiling (TLV-C) of 0.3 ppm until 2016; the value was then dropped to a Time Weighted Average (TLV-TWA) of 0.1 ppm (0.120 mg/m^3^) and a Short Term Exposure Limit (TLV-STEL) of 0.3 ppm (0.370 mg/m^3^). Conversely, the European Scientific Committee on Occupational Exposure Limits recently suggested a FA-related TWA of 0.3 ppm, but a STEL of 0.6 ppm (0.740 mg/m^3^)^8^.

Formalin is an aqueous solution usually containing 37–40% by weight of dissolved FA: its easy preparation and low cost make this compound the main cytological fixative in pathology laboratories worldwide^9,10^. Despite these advantages, the health and safety risks associated with formalin use are currently a matter of concern and FA toxicity is nowadays the main issue for its abolition in pathology laboratories^3,10,11^. Moreover, chronic exposures to FA, such as those present in workplaces, are suspected to be related to genotoxic effects^9^.

The FA genotoxic effect in occupationally exposed workers is still debated. Cytogenetic outcomes, such as increased chromosomal aberrations (CAs) and micronucleated cells (MNc), were reported in some bio-monitoring studies^12,13^ on chronic exposures, while this evidence was lacking in other published reports^14,15^.

However, the genomic damage level due to occupational exposure to xenobiotics depends also on the individual susceptibility. From the genetic point of view, this is due to polymorphisms in a battery of genes, mainly involved in metabolic and DNA-repair pathways^16^.

Phase I metabolic enzymes mostly consist of the cytochrome P450 (CYP) superfamily of microsomal enzymes^17^ catalysing oxidative reactions^18^, while phase II enzymes, such as glutathione S-transferases (GSTs), role is to increase the hydrophilicity of the xenobiotic compounds through conjugation reactions^17^. FA is quickly detoxified in the nasal tissues by oxidative reactions catalysed by glutathione-dependent and independent dehydrogenases, primarily the alcohol dehydrogenase 5^19^.

In order to safeguard the genome’s integrity and to prevent the potentially mutagenic consequences of DNA modifications, the cells evolved several mechanisms of DNA repair, according to the type of damage. The Base Excision Repair (BER) and the Nucleotide Excision Repair (NER) correct DNA small base changes (oxidation or alkylation) and bulky adducts, pyrimidine dimers and inter-strand cross-links, respectively^20^. These DNA-repair genes, which are involved in the protection mechanism against cancer development, are polymorphic^20^. Several evidences reported that defects in these DNA repair mechanisms could reduce FA tolerance at cellular level^21^.

Finally, several lines of evidence recently showed the FA role as oxidative stress inductor^3,22^. This imbalance between the production of Reactive Oxygen Species (ROS) and the capacity of the antioxidant system to counteract them, leads to biomolecular damages triggering inflammation, testified by massive proinflammatory cytokine release^23^, which is in turn related to carcinogenesis. Some cytokine gene polymorphisms, moreover, were found to modulate the amount of genomic damage associated with inflammatory and cancer diseases. As example, *TNF-α*, *IL-2*, *IL-6,* and *TGF-β1* polymorphisms have been showed to influence CAs level in cultured human peripheral blood lymphocytes (PBL)^24–26^. Despite of all these lines of evidence, the role of cytokine gene polymorphisms in modulating the FA exposure associated damage has not been completely clarified yet^26^.

In order to better elucidate the chronic FA genotoxic effect, we evaluated CAs frequency in PBL of pathologists chronically exposed to low air-FA concentration. This allows the detection of cells carrying unstable aberrations (*i.e.* chromosome and chromatid breaks, fragments) leading, in turn, to cell death during proliferation^27^. An increased CAs frequency in PBL is, thus, a powerful predictor of cancer risk significantly associated with the early events of carcinogenesis, as confirmed by previous studies in literature^28^. In order to evaluate the individual susceptibility role, sampled subjects were assessed for phase I, phase II, and DNA-repair gene polymorphisms, involved in the biotransformation, inactivation, and the DNA-repair processes, respectively. We analysed the most studied cancer-associated gene polymorphisms^29,30^, namely Cytochrome P450 1A1 (*CYP1A1*) exon 7 (A>G) *CYP1A1 2A* (T>C), CYP2C19*2 (G>A), *GSTT1*, *GSTM1*, *GSTP*, X-ray repair cross-complementing group 1 (*XRCC1*) 399 (G>A), 194 (C>T), 280 (A>G), Xeroderma pigmentosum complementation group C (*XPC*) exon 15 (A>C), XPC exon 9 (C>T) and Xeroderma pigmentosum complementation group D (*XPD*) (A>C). Finally, since cytokines play a fundamental role in the inflammatory process leading to genomic damage^26^, we assessed polymorphisms in *TNF-α* (− 308, G>A), *IL-10* − 1082 (G>A), *IL-10* (− 819, C>T), *IL-6* (− 174, G>C) as well.

The aim of the present study is thus to evaluate the role of chronic occupational FA exposure risk levels and the role of some genetic polymorphisms as possible modulators of genotoxic effects, in workers chronically exposed to low air-FA concentrations.

## Results

The epidemiologic sample includes 57 pathologists and 48 controls. In Table 1 are reported the demographic characteristics of the study population and the measured air-FA concentration on the sampling day. As expected, pathologists turned out to be exposed to an air-FA concentration significantly higher than controls (p < 0.001). No significant differences were found, instead, between the two groups concerning confounding factors such as sex, age, smoking habits and years of employment.

**Table 1:** Demographic characteristics and air-FA exposure level of subjects belonging to the studied groups.

	Pathologists (n = 57)	Controls (n = 48)
**Sex (n)**		
Males	29	25
Females	28	23
**Age (years)**		
Mean ± S.D.	42.632 ± 8.778	40.208 ± 9.711
Range	25–58	25–70
**Smokers (n)**	14	9
Number of cigarette/day (mean ± S.D.)	12.857 ± 10.939	13.667 ± 4.272
Years of smoking habit (mean ± S.D.)	19.643 ± 8.554	16.111 ± 7.688
**Non-smokers (n)**	43	39
**Years of employment (years)**		
Mean ± S.D.	11.246 ± 7.886	12.125 ± 7.482
Range	1–33	2–32
**Air-FA (μg/m**^**3**^**)**		
Mean ± S.D.	64.197 ± 32.385*	19.065 ± 5.173

*n* number of analysed subjects, *S.D.* standard deviation.

* P < 0.001, Kruskal–Wallis, Significantly higher with respect to Controls.

**Figure 1:**
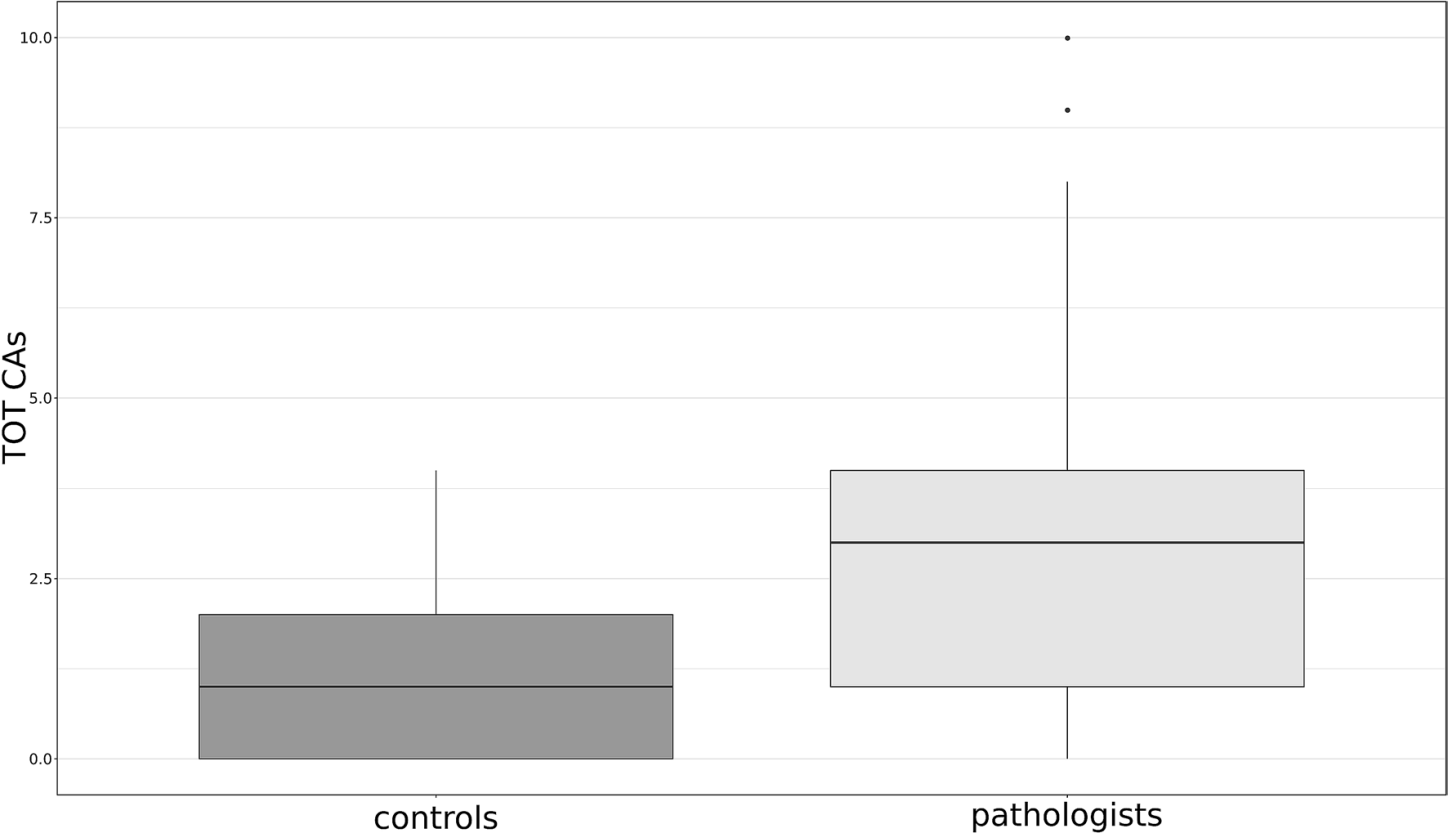
CAs frequency in exposed and control groups.

In Table 2 the level of genotoxic damage in the two sample groups is reported. We found three types of aberrations: chromatid break, chromosome break and acentric fragment. As can be seen, when compared to the control group, pathologists showed higher CAs and Ab.C frequencies (p < 0.001). The difference in CAs frequency between exposed and controls subjects is shown in Fig. 1.

**Table 2:** Frequencies of chromosomal aberrations and cells with aberrations in metaphases of lymphocytes from studied subjects.

Groups	N	NSM	B′	B″	AF	Total CAs	Total Ab.C	CAs/NSM % mean ± S.D	Ab.C/NSM % mean ± S.D
**Pathologists**	57	11,400	125	29	28	182	181	0.016 ± 0.012*	0.015 ± 0.011*
Males	29	5800	77	15	19	111	110	0.019 ± 0.013 A	0.019 ± 0.013 A
Females	28	5600	48	14	9	71	71	0.013 ± 0.010	0.013 ± 0.010
Smokers	14	2800	38	5	3	46	46	0.016 ± 0.015	0.016 ± 0.015
Non-smokers	43	8600	87	24	25	136	135	0.016 ± 0.011	0.016 ± 0.011
**Controls**	48	9600	42	9	18	69	68	0.007 ± 0.006	0.007 ± 0.006
Males	25	5000	14	5	8	27	26	0.005 ± 0.006	0.005 ± 0.006
Females	23	4600	28	4	10	42	42	0.009 ± 0.005 B	0.009 ± 0.005 B
Smokers	9	1800	5	2	3	10	9	0.006 ± 0.004	0.005 ± 0.004
Non-smokers	39	7800	37	7	15	59	59	0.008 ± 0.006	0.008 ± 0.006

*N* number of analysed subjects, *NSM* number of scored metaphases, *B*′ chromatid breaks, *B*″ chromosome breaks, *AF* acentric fragments, *CAs* chromosome aberrations, *Ab.C* cells with aberrations, *S.D.* standard deviation.

*P < 0.001, Kruskal–Wallis, significantly higher with respect to Controls.

A P = 0.046, Kruskal–Wallis, significantly higher with respect to Females.

B P = 0.020, Kruskal–Wallis, significantly higher with respect to Males.

The analyses of correlations performed on the whole sample showed a significant positive correlation between age and years of employment (r = 0.83, p < 0.001), CAs and Ab.C frequencies (r = 1.00, p < 0.001), CAs frequency and air-FA concentration (r = 0.33, p < 0.001) and, lastly, Ab.C frequency and air-FA concentration (r = 0.33, p < 0.001).

Multiple linear regressions were carried out to investigate the influence of the genetic profile in CAs frequency.

**Figure 2:**
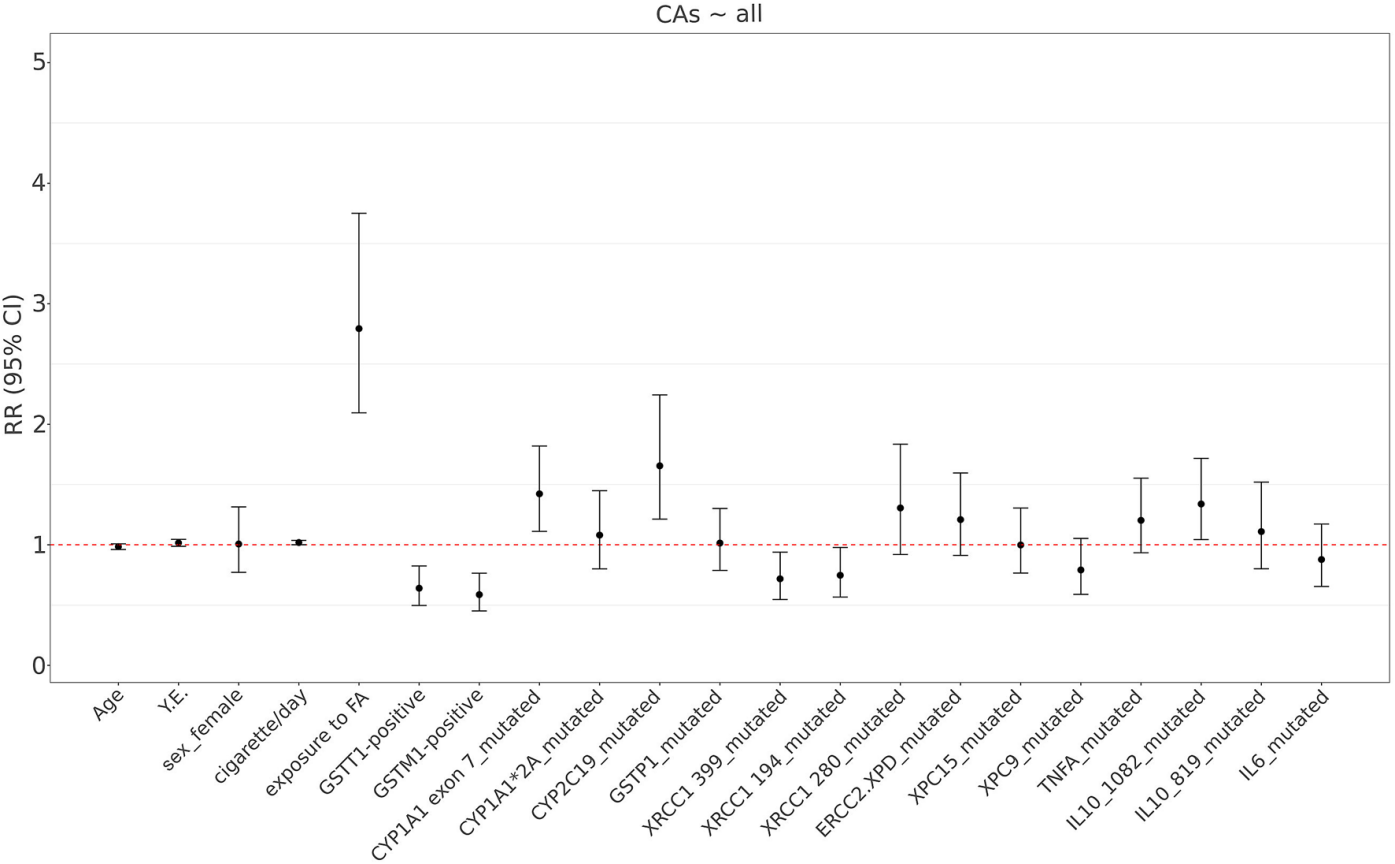
RR (95% CI) of developing CAs according to the various mutated allelic variants of genes considered in the present study, FA exposure and confounding factors as age, years of exposure, sex and cigarette/day (Model M0).

The model (Model M0) includes all genetic polymorphisms (wt vs carriers of at least one mutated allele) and confounding factors. There was a significant relationship between CAs frequency and exposure to air-FA (β = 1.027; p < 0.001), *CYP1A1 exon 7 (A*>*G)* (β = 0.353; p = 0.019), CYP2C19*2 (G>*A)* (β = 0.504; p = 0.007), *GSTT1*-positive (β = − 0.447; p = 0.004), *GSTM1*-positive (β = − 0.533; p = 0.001) and *XRCC1 (399, G*>*A)* (β = − 0.331; p = 0.044) genotypes. A tendency in increasing CAs frequency, albeit not significant, was found for *IL-10 1082 (G*>*A)* genotype (β = 0.292; p = 0.054). Figure 2 shows the Relative Risk (RR) of developing CAs according to the presence of mutated allelic variants of the gene considered.

## Discussion

Despite the growing awareness regarding the harmful effects of air-FA exposure, FA is currently employed in hospital pathology laboratories raising concerns about pathologists safety^31,32^.

Many studies demonstrated the FA genotoxicity both in vitro and in vivo, considering various biological systems and endpoints^5^. Moreover, occupational and environmental exposures are often chronic and mixed, and the analysis of their outcomes should take into account also the eventuality of a cumulative genomic instability induced by chronic exposures^33^.

As expected, we found significantly higher CAs and Ab.C frequencies in pathologists than in controls, in agreement with literature evidence, even though conflicting results can be found^12,34,35^. In this regard, Costa et al.^34^ reported that even at an average FA concentration of 0.38 ppm (i.e., 0.47 mg/m^3^) frequencies of cytogenetic parameters, such as CAs, were significantly higher in pathologists than in controls. In order to explain the mechanisms leading to FA-induced genotoxicity, several hypotheses have been proposed: DNA–protein cross-links, damage to proteins required for the mitotic process and reduced expression of paxillin, an essential component of the abscission machinery required to complete cytokinesis, which may lead, respectively, to DNA replication stress and DNA breaks, chromosome malsegregation during nuclear division and cytokinesis failure leading to micronucleus formation and aneuploidy^7^. Moreover, the inflammatory process, due to the activation of neutrophils and eosinophils and/or the altered redox balance in the bone-marrow could play a central role in DNA strand breaks induced by ROS^7^.

The harmful effects of xenobiotics exposure is extremely shaped by individual susceptibility. The analysis of metabolic and DNA-repair gene polymorphisms in risk assessment of hazardous chemicals assume thus particular importance^5,26^.

In this context, we focused on the role of several genetic polymorphisms in modulating CAs frequency in subjects occupationally exposed to air-FA compared to a control group.

We found a significant effect of some genetic polymorphisms in CAs frequency modulation, even among genes coding for enzymes not directly involved in FA metabolism.

Specifically, among genes coding for phase I metabolism enzymes, we found a significant increase in CAs frequency in carriers of *CYP1A1* exon 7 (A>G) and CYP2C19*2 (G>A) polymorphisms. These genes are members of the cytochrome P450 superfamily of enzymes, mixed-function mono-oxygenases responsible for metabolizing, mainly via oxidative reactions, several exogenous and endogenous compounds, including steroids, fatty acids, retinoid, drugs, vitamins, procarcinogens/promutagens, and environmental compounds^36,37^.

The *CYP1A1* Ile462Val substitution in the heme-binding domain of exon 7, leads to a concurrent increase in the catalytic activity of the protein and was associated with lung cancer risk^38,39^. The Ile/Ile genotype was also found to be associated with an increase of aberrant cells, and to be a CAs predictor^40^. Contrary, other studies did not find any association^41^.

Common variants of the *CYP2C19* gene are associated with impaired drug metabolism. CYP2C19*2 results from a guanine (G) to adenine (A) transition at position 681 in exon 5, producing an aberrant splicing site and encoding enzymes with decreased activity^42^. This polymorphism was related to genotoxicity in a previous study of Santovito et al., where CYP2C19 A/A subjects turned out to show a frequency of sister chromatid exchanges (SCEs) significantly higher with respect to the CYP2C19 G/G homozygote genotypes^43^.

In phase II enzymes, we found a significant CAs frequency decrease in *GSTT1*-positive and *GSTM1*-positive subjects. Accordingly, the higher frequency of genotoxic damage in carriers of the null-allele could be explained considering the role of these genes and their mutations on metabolism. The glutathione S-transferases represent an important group of enzymes, which detoxify both endogenous and exogenous compounds, included pharmaceuticals and environmental pollutants^44^. The *GSTM1* and *GSTT1* polymorphisms consist both in the deletion of a part of the gene, leading, in homozygous individuals, to a lack of the enzyme activity^45^. In literature, the *GSTM1*-null and *GSTT1*-null genotypes have been related to increased risk for several cancers, such as lung and colorectal cancer^45^. These are enzymes directly involved in the FA metabolism. Due to its high-water solubility and reactivity, indeed, airborne FA is absorbed mainly (~ 90%) in the upper respiratory tract, where it quickly forms intermolecular and intramolecular cross-links within proteins and nucleic acids at the site of contact. It is also rapidly metabolized to formate by FA-dehydrogenase requiring glutathione: the depletion of this compound in the absorbing tissues results in more FA bound to DNA within cells^46,47^. While some reports showed no effect of these polymorphisms in modulating the level of genomic damage^12,34,45,46^, others found a significant association. As example, Santovito et al.^43^ observed higher frequencies of SCEs, CAs, and Ab.C among pathologists with *GSTT1*-null genotypes than in the reference group. Several other epidemiological studies evaluating exposure to organic solvents, reported the *GSTM1*-null genotype associated with an increase in cytogenetic biomarkers, probably due to the absence of detoxification activity that may affect the amount of DNA damage^48,49^.

Since DNA damage is a key step in the carcinogenic process^50^, we also considered polymorphisms in both BER and NER pathways. Unexpectedly, we found a significant relationship only for *XRCC1 (399, G*>*A)* polymorphism, which turned out to be related to a reduction in CAs frequency. The X-ray cross-complementing group 1 (*XRCC1*) is a major DNA repair gene involved in base BER, which is able to fix DNA base damage and single-strand breaks through interacting with DNA components at the damage site. The polymorphisms related to this gene have been linked to the development of several types of cancer^51,52^. Specifically, the *XRCC1* Arg399Gln polymorphism has been reported to reduce the oxidative damage repair activity and the 399Gln allele has been shown to be related to higher mutagen sensitivity and higher levels of DNA adducts^53^. Therefore, our result appears to be inconsistent with the gene function. Nevertheless, in literature contrasting results can be found. In workers exposed to organic solvents, Hoyos-Giraldo et al. reported that the *XRCC1* Arg399Gln polymorphism carriers did not have a significant CAs frequency increase compared to the wild type genotype carriers. Contrary, in benzene-exposed workers, a significant CAs frequency increase related to by *XRCC1* Arg399Gln variant was reported^48^. As well, in a study on active and passive smokers, Gln/Gln carriers reveal a significantly higher number of aberrations than the Arg/Gln and Arg/Arg genotypes in both the controls and exposed subjects^54^.

No significant relationship was found between CAs frequency and *XRCC1* (280, A>G) polymorphism, even though in literature this association has been reported^48^.

Finally, inflammation and oxidative stress are knowingly interdependent pathophysiological processes^55^. Since these are two possible mechanisms through which FA could explain its harmful effects, we evaluated the role of polymorphisms on both pro- and anti-inflammatory cytokines genes. No significant result was found, according to the study of Santovito et al.^26^, which found no association between cytogenetic damage and *TNFα* − 308 (G>A), *IL10* − 1082 (G>A) and *IL10* − 819 (C>T) gene polymorphisms, with the only exception of homozygous genotypes for *IL-6* G allele, that showed a significant decrease in the frequency of SCEs compared to heterozygous subjects.

These results, however, should be considered cautiously, as we did not consider the eventual effect of all the possible confounding factors that could modulate the studied outcomes, such as the plethora of chemicals to whom pathologists could be exposed in various degrees in laboratories.

## Conclusions

Our study confirms FA genotoxic effect, even in workers chronically exposed to low FA levels. Several genetic polymorphisms in metabolism and DNA-repair pathways seem to have an influence in modulating the effect of FA exposure. These findings further highlight the importance of individual susceptibility biomarkers assessment in occupational studies. Due to the extreme FA widespread presence in environmental and occupational settings, studies on both harmful effects related to FA exposure and modulators are crucial to elaborate effective Public Health preventive strategies.

## Materials and methods

### Epidemiological sample

The epidemiological sample consist of 57 workers occupationally exposed to FA enrolled in two pathology wards of Turin (Italy) and forty-eight hospital workers not exposed to FA recruited in the same two hospitals as control group. Each volunteer signed an informed consent form. The sampling was performed on Wednesday of each sampling week, engaging five to eight subjects every time. Since, routinely, the exposure to FA in pathology wards occurs mainly via inhalation, each participant wore a personal passive sampler for the measurement of air-FA concentration during the sampling-day work shift. At 4 p.m. of the same day, each subject provided a venous blood sample and answered to a questionnaire administered by one interviewer. The study was approved by the Bioethical Committee of the University of Turin and was performed in accordance with the ethical standards laid down in the 2013 Declaration of Helsinki.

### Questionnaire

The questionnaire was administered to each subject by an interviewer to obtained information about demographic characteristics (sex, age), personal habits (smoking) during the last year, and work characteristics (length in years of service working and type of work).

### Personal air-FA collection and analysis

FA air samples were collected for working shift (8 h) on Wednesday using passive personal air samplers clipped near the breathing zone of the subject, according to Santovito et al.^12^.

### Blood sample collection and chromosomal aberration analysis

Blood sample collection and chromosome aberration analysis were performed according to Santovito et al.^12^. DNA extraction and genotyping procedure were carried out as described in Ruberto et al.^56^. Primer sequences, melting temperatures, PCR methodologies used, and expected PCR product sizes are reported in Supplementary Table S1 online.

### Statistical analyses

Statistical analysis was assessed using the SPSS software statistical package programme (version 22.0, Chicago, USA) and R (R version 4.0.2). Differences between sex, mean age and years of employment (y.e.) among and between groups were evaluated by analysis of variance. A non-parametric Kruskal–Wallis test was used to compare age, mean y.e. and CAs frequency between groups.

Multivariate general linear model, with Bonferroni’s correction, was used to evaluate the influence of age and years of exposure on CAs frequency in both groups. All p-values were two tailed and the level of statistical significance was set at p < 0.05 for all tests.

Association between both genetic and environmental variables with the level of genomic damage was evaluated by Poisson regression model, due to a Poisson nature and distribution of the dependent variable (CAs) (Supplementary Fig. S1 online). Genetic variables were considered both in binarized (wild type vs. any mutated allele) and multiallelic (wild type vs. heterozygous vs. homozygous).

## Supplementary Information

Supplementary Information.

## Data Availability

The datasets generated during and/or analysed during the current study are available from the corresponding author on reasonable request.
