# Supplementary material for: The role of phase I, phase II, and DNA-repair gene polymorphisms in the damage induced by formaldehyde in pathologists
Source: Sci Rep. 2021 May 18;11:10507. doi: 10.1038/s41598-021-89833-w (PMC8131755; doi:10.1038/s41598-021-89833-w)
Supplement: Supplementary file 1 — Supplementary Information. [file 41598_2021_89833_MOESM1_ESM.pdf]

**Supplementary Materials**

**Title:**
The role of phase I, phase II, and DNA-repair gene polymorphisms in the damage induced by formaldehyde in pathologists.

**Authors:**
Federica Ghelli[1], Enrico Cocchi[1], Martina Buglisi[1], Giulia Squillacioti[1], Valeria Bellisario[1], Roberto Bono[1*†], Alfredo Santovito[2†]
[†] These authors contributed equally to this work as last author

**Affiliations:**
[1] Department of Public Health and Pediatrics, University of Turin, via Santena 5 bis, Turin, 10126, Italy
[2] Department of Life Sciences and Systems Biology, University of Turin, Via Accademia Albertina 13, Turin, 10123, Italy

**Corresponding Author:**
*Prof. Roberto Bono, Department of Public Health and Pediatrics, University of Turin, Italy. Via Santena 5 bis, 10126 Turin, Italy

E-mail address: roberto.bono@unito.it

**Supplementary Table S1** Primers, annealing temperatures and methodologies used for the analysed gene polymorphisms

| Gene | Sequence | T (°C) | Methodology | PCR product size (bp) | Reference |
|---|---|---|---|---|---|
| *CYP1A1 exon 7* | 5′- AAGACCTCCCAGCGGGCAAT - 3′<br>5′- AAGACCTCCCAGCGGGCAAC - 3′<br>5′- CTCTGGTTACAGGAAGCTAT - 3′ | 60 | PCR | 162 | 1 |
| *CYP1A1*2A* | 5’- CAGTGAAGAGGTGTAGCCGCT - 3’<br>5’- TAGGGAGTCTTGTCTCATGCCT - 3’ | 60 | RFLP (MaspI) | PCR product: 340 bp<br>T-allele = 340 bp<br>C-allele = 200 and 140 bp | 2 |
| *CYP2C19*2* | 5’- CAGAGCTTGGCATATTGTATC - 3’<br>5’- TATCGCAAGCAGTCACATAAC- 3’<br>5'-ACTATCATTGATTATTTCCCG-3’<br>5’-GTAATTTGTTATGGGTTCCT-3’ | 57 | ARMS-PCR | PCR Product: 373 bp<br>2*G-allele = 283 bp<br>2*A-allele = 129 bp. | 3 |
| *GSTT1* | 5’- TTCCTTACTGGTCCTCACATCTC - 3’<br>5’- TCACCGGATCATGGCCAGCA - 3’ | 63 | PCR | 480 | 4 |
| *GSTM1* | 5- CTGGATTGTAGCAGATCATGC - 3’<br>5’- CTGCCCTACTTGATTGATGGG - 3’ | 65 | PCR | 273 | 5 |
| *GSTP* | 5’- AATACCATCCTGCGTCACCT - 3’<br>5- TGAGGGCACAAGAAGCCCCTT - 3’ | 60 | RFLP (BsmA I) | PCR Product: 566 bp<br>A-allele = 308 + 258 bp<br>G-allele = 258 + 219 + 89 bp | 6 |
| *XRCC1 (399, G>A)* | 5’- CAAGTACAGCCAGGTCCTAG - 3’<br>5’-CCTTCCCTCATCTGGAGTAC - 3’ | 60 | RFLP (Nci I) | PCR Product: 248 bp<br>G-allele = 159 + 89<br>A-allele = 248 bp | 7 |
| *XRCC1 (194, C>T)* | 5’- GCCCCGTCCCAGGTA – 3’<br>5’- AGCCCCAAGACCCTTTCACT - 3’ | 60 | RFLP (MspI) | PCR Product: 383 bp<br>C-allele = 346 + 37 bp<br>T-allele = 383 bp | 8 |
| *XRCC1A (280, A>G)* | 5’-TGGGGCCTGGATTGCTGGGTCTG – 3’<br>5’- CAGCACCACTACCACACCCTGAAGG – 3’ | 60 | RFLP (RsaI) | PCR Product: 280 bp<br>A-allele = 140 bp<br>G-allele = 280 bp | 8 |
| *ERCC2/XPD (751, A>C)* | 5’ – TCAAACATCCTGTCCCTACT- 3’<br>5’ – CTGCGATTAAAGGCTGTGGA – 3’ | 58 | RFLP (Pst I) | PCR Product: 344 bp<br>A-allele = 234+110 bp<br>C-allele = 171+110+63 bp | 9 |
| *XPC exon 15 (A>C)* | 5’ – ACCAGCTCTCAAGCAGAAGC – 3’<br>5’ – CTGCCTCAGTTTGCCTTCTC – 3’ | 60 | RFLP (Pvu II) | PCR Product: 281 bp<br>A-allele = 281<br>C-allele = 150+131 bp | 10 |
| *XPC exon 9 (C>T)* | 5’ – TAAGGACCCAAGCTTGCCCG – 3’<br>5’ – CCCACTTTTCCTCCTGCTCACAG – 3’ | 60 | RFLP (Sac II) | PCR Product: 152 bp<br>C-allele = 131+21 bp<br>T-allele = 152 bp | 10 |
| *TNF-α (-308, G>A)*<br>- Antisense primer<br>- G-sense primer<br>- A-sense primer | 5’-TCTCGGTTTCTTCTCCATCG-3’<br>5’-ATAGGTTTTGAGGGGCATGG-3’<br>5-AATAGGTTTTGAGGGGCATGA-3’ | 60 | ARMS-PCR | 184 | 11 |
| *IL 10 -1082 (G>A)*<br>- Antisense primer<br>- G-sense primer<br>- A-sense primer | 5’-AGTGCCAACTGAGAATTTGG-3’<br>5’-CTACTAAGGCTTCTTTGGGAG-3’<br>5’-ACTACTAAGGCTTCTTTGGGAA-3’ | 60 | ARMS-PCR | 258 | 11 |
| *IL-10 (-819, C>T)*<br>- Antisense primer<br>- C-sense primer<br>- T-sense primer | 5’-AGGATGTGTTCCAGGCTCCT-3’<br>5’-CCCTTGTACAGGTGATGTAAC-3’<br>5’-ACCCFFGTACAGGTGATGTAAT-3’ | 60 | ARMS-PCR | 233 | 11 |
| *IL-6 (-174, G>C)*<br>- Antisense primer<br>- G-sense primer<br>- C-sense primer | 5’-TCGTGCATGACTTCAGCTTTA-3’<br>5’-AATGTGACGTCCTTTAGCATG-3’<br>5’-AATGTGACGTCCTTTAGCATC-3’ | 60 | ARMS-PCR | 190 | 12 |


1. Chen, S., Xue, K., Xu, L., Ma, G. & Wu, J. Polymorphisms of the CYP1A1 and GSTM1 genes in relation to individual susceptibility to lung carcinoma in Chinese population. *Mutat. Res. - Mutat. Res. Genomics* **458**, 41–47 (2001).

2. Salehi, Z., Gholizadeh, L., Vaziri, H. & Madani, A. H. Analysis of GSTM1, GSTT1, and CYP1A1 in Idiopathic Male Infertility. *Reprod. Sci.* **19**, 81–85 (2012).

3. Bonello, L. *et al.* Clopidogrel Loading Dose Adjustment According to Platelet Reactivity Monitoring in Patients Carrying the 2C19*2Loss of Function Polymorphism. *J. Am. Coll. Cardiol.* **56**, 1630–1636 (2010).

4. Pemble, S. *et al.* Human glutathione S-transferase Theta (GSTT1): cDNA cloning and the characterization of a genetic polymorphism. *Biochem. J.* **300**, 271–276 (1994).

5. Zhong, S., Wyllie, A. H., Barnes, D., Wolf, C. R. & Spurr, N. K. Relationship between the gstm1 genetic polymorphism and susceptibility to bladder, breast and colon cancer. *Carcinogenesis* **14**, 1821–1824 (1993).

6. García-González, M. A. *et al.* Relevance of GSTM1, GSTT1, and GSTP1 gene polymorphisms to gastric cancer susceptibility and phenotype. *Mutagenesis* **27**, 771–777 (2012).

7. Matullo, G. *et al.* XRCC1, XRCC3, XPD gene polymorphisms, smoking and 32P-DNA adducts in a sample of healthy subjects. *Carcinogenesis* **22**, 1437–1445 (2001).

8. Wang, Q. *et al.* Genetic polymorphisms of XRCC1, HOGG1 and MGMT and micronucleus occurrence in Chinese vinyl chloride-exposed workers. *Carcinogenesis* **31**, 1068–1073 (2010).

9. Li, Y. *et al.* Gene-environment interactions between DNA repair polymorphisms and exposure to the carcinogen vinyl chloride. *Biomarkers* **14**, 148–155 (2009).

10. Hu, Z. *et al.* DNA repair gene XPC genotypes/haplotypes and risk of lung cancer in a Chinese population. *Int. J. Cancer* **115**, 478–483 (2005).

11. Perrey, C., Turner, S. J., Pravica, V., Howell, W. M. & Hutchinson, I. V. ARMS-PCR methodologies to determine IL-10, TNF-α, TNF-β and TGF-β1 gene polymorphisms [2]. *Transplant Immunology* vol. 7 127–128 (1999).

12. Zakharyan, R. *et al.* Interleukin-6 promoter polymorphism and plasma levels in patients with schizophrenia. *Tissue Antigens* **80**, 136–142 (2012).

**CAs distribution plot**

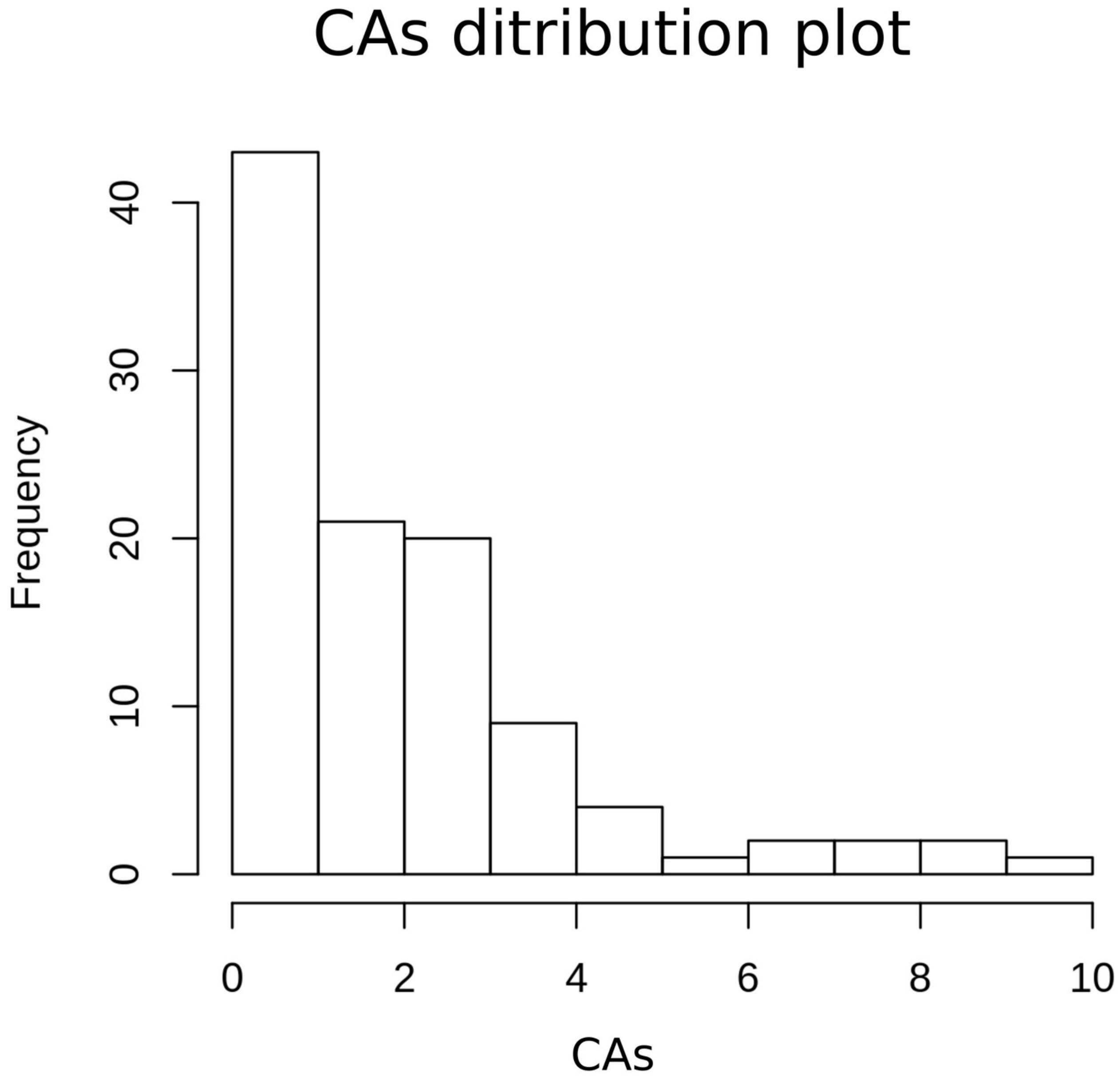


**Supplementary Figure S1** Poissonian distribution of CAs in the sampled population
